# Cherenkov Luminescence Imaging for Assessment of Radioactive Plaque Position in Brachytherapy of Uveal Melanoma: An In Vivo Feasibility Study

**DOI:** 10.1167/tvst.9.7.42

**Published:** 2020-06-29

**Authors:** Jørgen Krohn, Yi-Chun Chen, Nils Ole Stabo-Eeg, Børge Hamre

**Affiliations:** 1 Department of Clinical Medicine, Section of Ophthalmology, University of Bergen, Bergen, Norway; 2 Department of Ophthalmology, Haukeland University Hospital, Bergen, Norway; 3 Department of Physics and Technology, University of Bergen, Bergen, Norway

**Keywords:** cherenkov luminescence imaging, episcleral brachytherapy, ruthenium-106 plaque, uveal melanoma

## Abstract

**Purpose:**

To study the feasibility of using Cherenkov luminescence imaging (CLI) to evaluate and document ruthenium-106 plaque position during brachytherapy of uveal melanoma.

**Methods:**

Ruthenium-106 decays by emitting high-energy beta particles. When the electrons pass through the eye, Cherenkov radiation generates a faint light that can be captured by highly sensitive cameras. Patients undergoing ruthenium-106 plaque brachytherapy for posteriorly located choroidal melanoma were examined by CLI, which was performed in complete darkness with an electron multiplying charged-coupled device camera mounted on a fundus camera modified for long exposures.

**Results:**

Ten patients with tumors ranging from 5.8 to 13.0 mm in largest basal diameter and 2.0 to 4.6 mm in height were included. The plaques had an activity between 0.035 and 0.089 MBq/mm^2^ at the time of examination (1–4 days after implantation). CLI revealed the actual plaque position by displaying a circular area of light in the fundus corresponding with the plaque area. The Cherenkov light surrounded the tumor as a halo, which showed some asymmetry when the plaque was slightly displaced. The light intensity correlated positively with plaque activity and negatively with tumor pigmentation. Exposure times between 30 and 60 seconds were required to display the plaque position and delineate the tumor area. The long exposures made it difficult to maintain stable eye fixation and optimal image quality.

**Conclusions:**

CLI is a novel method to assess and document ruthenium-106 plaque position in brachytherapy for uveal melanoma.

**Translational Relevance:**

Ocular CLI may provide relevant radiation data during and after implantation of radioactive plaques, thus improving the accuracy of episcleral brachytherapy.

## Introduction

Episcleral brachytherapy is currently the most widely used conservative treatment for uveal melanoma.^1^ Accurate radiation dosimetry and precise placement of the radioactive plaque in relation to the tumor are the two most important factors to achieve local tumor control and to reduce the risk of radiation-induced side effects.

Various techniques have been applied both intraoperatively and postoperatively to ensure that the attached radioactive plaque covers the tumor base with the required tumor-free margin. During surgery, indirect ophthalmoscopy with transscleral transillumination and different forms of ultrasound examination are commonly used.^2^^–^^8^ Ultrasound examination is also the most common technique to assess plaque placement after surgery.^9^^–^^11^ Other methods include magnetic resonance imaging^12^^,^^13^ and the use of plaque-mounted light-emitting diodes.^14^^,^^15^

For posteriorly located choroidal melanomas, it can be challenging to position the plaque correctly, which may explain why radiation failure is more common in such cases.^16^ Furthermore, initially well-placed plaques may become displaced or tilted by eye movements, hemorrhages, adjacent extraocular muscles, the optic nerve sheath, or posterior ciliary nerves and vessels, which in turn can decrease the radiation dose to the tumor and cause local tumor recurrence.^11^^,^^17^ High-resolution ultrasound examination can be used to correctly determine plaque position during and after surgery, but it might be hampered by varying image resolution and imaging errors.^18^ Still, the development of new methods to assess and document plaque placement in episcleral brachytherapy is warranted.

Cherenkov luminescence imaging (CLI) is an optical imaging modality that relies on the Cherenkov radiation effect, where charged particles induce faint visible light when travelling faster than the speed of light through a dielectric medium.^19^ Ruthenium-106 is a frequently used isotope for episcleral brachytherapy of uveal melanoma. Ruthenium-106 decays with a half-life of 373.6 days via rhodium-106 to palladium-106. Rhodium-106 has a half-life of about 30 seconds and emits highly energetic electrons with a maximum energy of 3.5 MeV.^20^ Hence, the electrons from rhodium-106 have sufficient energy to induce Cherenkov radiation as they pass through the eyewall and further into the vitreous. This phenomenon has been shown in a recent in vitro study, where CLI made it possible to verify the position and tilting of ruthenium-106 plaques relative to melanin-containing tumor phantoms in enucleated porcine eyes.^20^ The emitted Cherenkov radiation varies over a wide range in the visible spectrum with a peak in the ultraviolet–blue region.^21^ In biological tissues, which favor the transmission of red–infrared light, the emitted photons are highly scattered and absorbed.^21^ The detection of Cherenkov light in tissue is, therefore, challenging and requires highly sensitive techniques, such as the use of electron multiplying charged-coupled device (EMCCD) cameras in completely dark environments. Herein, we demonstrate the feasibility of using CLI to assess and document the plaque position during ruthenium-106 brachytherapy in patients with posterior uveal melanoma.

## Methods

### Patients and Study Design

This prospective, clinical feasibility study was conducted at the Department of Ophthalmology at Haukeland University Hospital, which is one of two national referral centers for ocular oncology. The study was carried out between January 2018 and November 2019. Inclusion criteria were patients undergoing ruthenium-106 plaque brachytherapy for choroidal melanoma. The tumor had to be located so close to the posterior pole that it could be visible in fundus images within the limits of a 45° field of view. The patients also had to be cooperative with CLI during the early postoperative period. The decision as to which day should be selected for the examination was based on practical considerations and the patient's wishes and general condition. Tumor pigmentation was subjectively graded as weak, moderate, marked, or partial, based on ophthalmoscopy and fundus photography. The Cherenkov luminescence images were evaluated according to their quality and ability to demonstrate the plaque position relative to the tumor. Conventional (Zeiss FF450; Carl Zeiss Meditec, Jena, Germany) and ultra-widefield (California; Optos plc, Dunfermline, Scotland, UK) fundus photography were performed during follow-up to verify the actual plaque position based on the radiation induced chorioretinal scar formation.

In addition to the clinical CLI of uveal melanoma patients, experimental CLI of ruthenium-106 plaques placed in a water-filled container was performed to optimize instrumentation performances and to visualize the pattern of Cherenkov radiation generated in the water adjacent to the plaque.

This study was registered and approved by the Regional Committee for Medical and Health Research Ethics, Western Norway (reference number: 2015/552), and followed the official ethical regulations for clinical research and the Declaration of Helsinki. All patients gave their written informed consent before participation.

### Ruthenium-106 Plaques and Surgical Technique

The ruthenium-106 plaques used in the study were of the models CCA and CCB (Eckert & Ziegler BEBIG GmbH, Berlin, Germany). These plaques have two eyelets for surgical attachment and are circular with a diameter of 15.3 mm and 20.2 mm. The diameter of the radiation zone is slightly smaller than the diameter of the plaque itself, leading to an annular inactive rim of 0.75 mm. The mean plaque activity at the time of CLI was 9.9 MBq (range, 7.0−12.8 MBq) and 15.8 MBq (range, 10.4−21.8 MBq) for the CCA and CCB plaques, respectively. The prescription dose was 90 Gy to the tumor apex.

Surgery was performed under general anesthesia. Plaque implantation was guided by indirect ophthalmoscopy through a surgical microscope with slit-lamp attachment and a handheld contact lens (QuadrAspheric; Volk Optical Inc., Mentor, OH), combined with fiber optic transscleral transillumination. The plaque was secured to the sclera by two 7-0 silk sutures. Owing to the posterior tumor location, all plaques were positioned eccentrically (in the anteroposterior axis) relative to the melanoma to decrease the radiation dose to the fovea and optic disc. Therefore, the reported plaque displacement in our patients did not apply to the intended eccentric plaque location.

### Instrumentation

**Figure 1.**
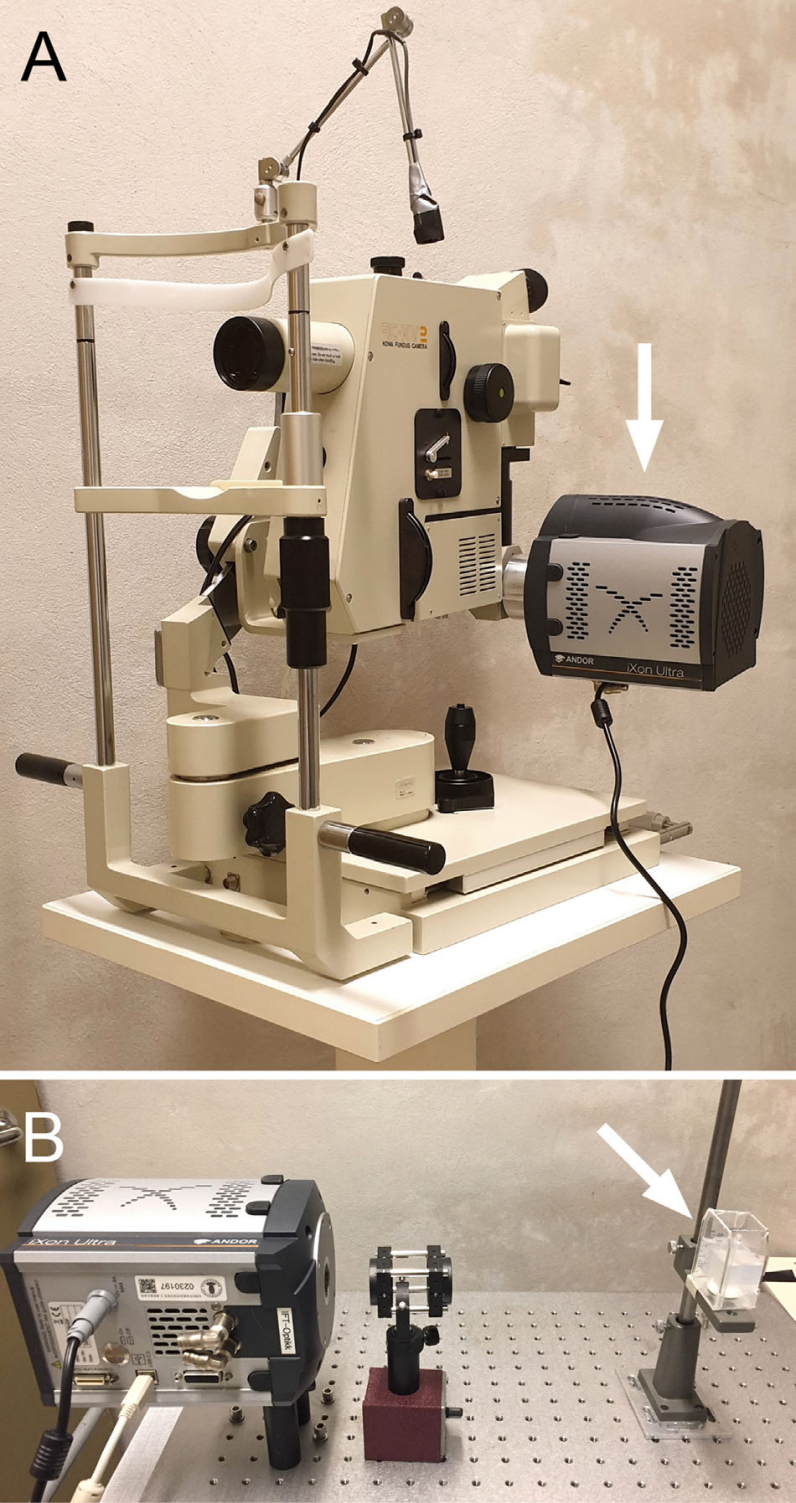
Photographs of the experimental setup. (A) The EMCCD camera (arrow) mounted on the fundus camera for clinical CLI of uveal melanoma patients. (B) The EMCCD camera and optical system used for experimental CLI of water-immersed ruthenium-106 plaques (arrow).

The clinical CLI of uveal melanoma patients was performed with an EMCCD camera coupled to a fundus camera. For the experiments conducted in 2018, the images were acquired with the EMCCD camera Andor iXon DV887 (Andor Technologies, Belfast, Ireland), which from 2019 was replaced by the upgraded Andor iXon Ultra 897. Both cameras have a back-illuminated 8.2 × 8.2 mm sensor with the capability of single photon detection and thermoelectric cooling down to –90°C to –100°C to optimize sensitivity and decrease dark current noise. The EMCCD camera was connected to a personal computer and attached to an analog 45° field of view fundus camera system (Kowa RC-XV2; Kowa, Tokyo, Japan) modified to take long exposures (Fig. 1A). The software Andor-SOLIS 64-bit (Oxford Instruments, Abingdon-on-Thames, UK) was used for image acquisition and storage.

For the experimental CLI of ruthenium-106 plaques, the EMCCD camera (Andor iXon Ultra 897) was focused on the plaque by means of two achromatic optical lenses with a focal length of 60 mm and 160 mm (Spindler & Hoyer, Gottingen, Germany), respectively, placed in front of the camera. The 60-mm lens was positioned one focal length from the plane of the sensor, which was determined through the camera's real-time video function. The 160-mm lens was placed one focal length from the plaque, and directly next to the 60-mm lens. The ruthenium-106 plaque (CCB) was put into a transparent acrylic box filled with distilled water, and placed at different positions and orientations relative to the EMCCD camera (Fig. 1B).

To eliminate any ambient light that would otherwise lead to overexposure of the images, the CLI was carried out in total darkness. This was achieved by placing the instrumentation in a secluded part of the hospital basement and keeping the fundus/EMCCD camera and the connected computer in separate rooms. For the same reason, the patient fixation light, which consisted of a red light-emitting diode (LED) with a peak wavelength of about 680 nm, was either dimmed to a minimum or turned off during the imaging process. For the clinical CLI conducted from 2019 onward, a 600-nm short-pass filter (FES0600; Thorlabs Inc., Newton, NJ) was inserted into the EMCCD mounting bracket directly in front of the camera sensor to minimize the amount of ambient light from the LED and thereby optimize eye fixation.

### Image Processing

Before the experimental and clinical CLI, a reference image of the ruthenium-106 plaque and fundus, respectively, was acquired with a similar instrument setup and by operating the EMCCD without additional signal amplification in low light conditions with exposure times between 0.1 and 0.001 seconds.

The image acquisition settings for the clinical CLI of patients with uveal melanoma were altered between a few fixed values depending on various parameters such as plaque activity, degree of tumor pigmentation, and the patient's ability to cooperate during CLI. The exposure time was set to 30, 45, or 60 seconds, mainly based on plaque activity (i.e., the weaker the radioactivity, the longer the exposure time). A hardware pixel binning of 2 × 2 or 4 × 4 was used to optimize the sensitivity and signal-to-noise ratio. To achieve sufficient image contrast, the electron multiplying and pre-amplifier gains were set to 250× and 3×, respectively.

## Results

### Experimental CLI of Ruthenium-106 Plaques

**Figure 2.**
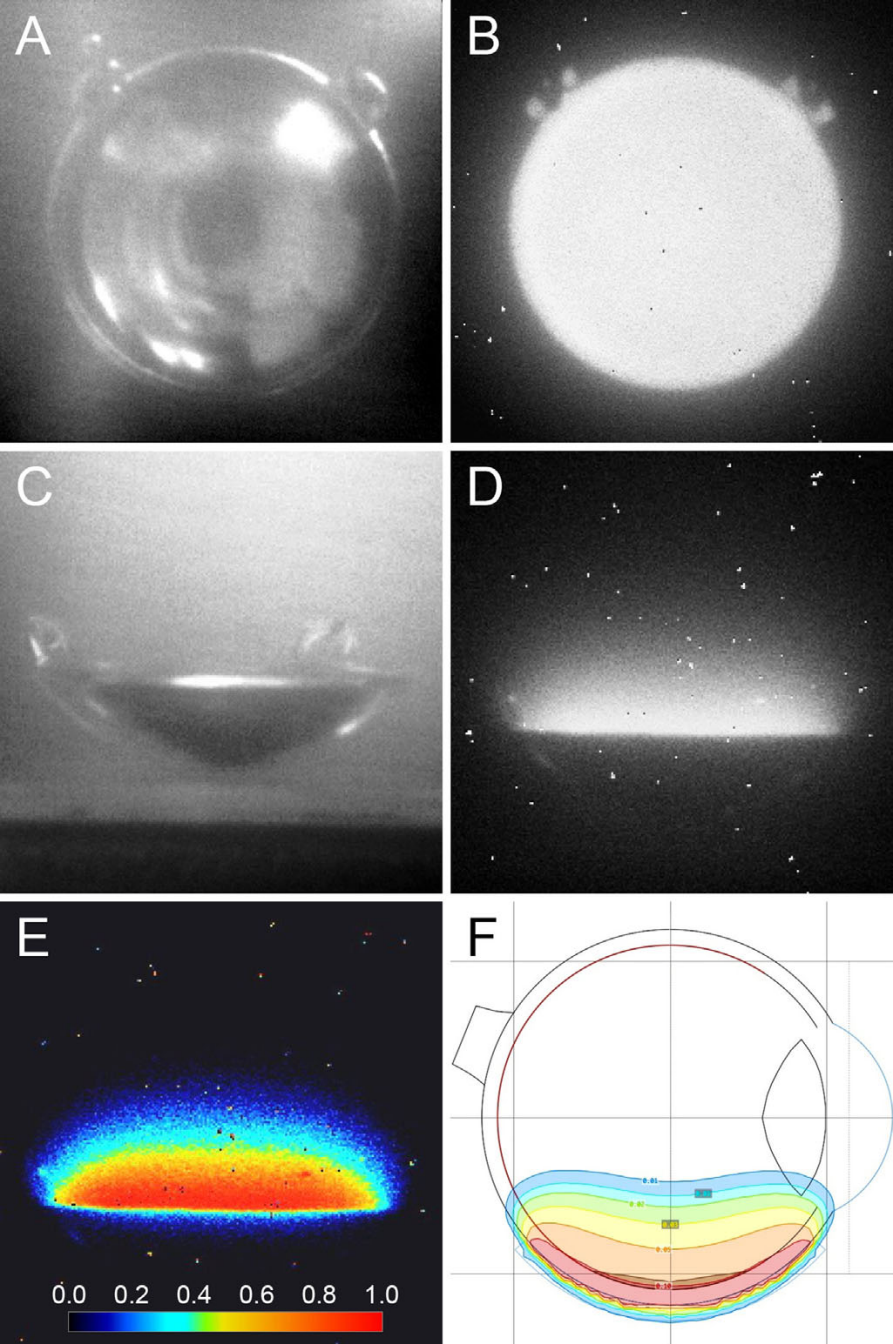
Experimental CLI of the ruthenium-106 plaques. (A) Front view reference image, taken in low light conditions, of the concave radioactive surface of a CCB plaque immersed in distilled water. (B) Cherenkov luminescence image of the same plaque as shown in (A) taken in total darkness with an exposure time of 30 seconds. Note the homogenous emission of Cherenkov light spreading slightly beyond the plaque perimeter. (C) Side view reference image, taken in low light conditions, of a CCB plaque immersed in distilled water. (D) Cherenkov luminescence image of the same plaque as shown in (C) taken in total darkness with an exposure time of 60 seconds. Note the dome-shaped, luminous area reaching slightly beyond the rim and 8–10 mm above the edge of the plaque. (E) False color-coded version of the same image as shown in (D). The colors indicate the relative light intensity, as given by the color scale at the bottom. (F) Two-dimensional dose distribution diagram of a CCB plaque with the same activity and radiation time as the plaque shown in (D, E).

Two different setups were used to image the pattern of Cherenkov radiation from the water-immersed ruthenium-106 plaques. First, a CCB plaque with an activity of 19.9 MBq was placed vertically to enable front view imaging of its concave radioactive surface (Fig. 2A). CLI of the plaque in this position revealed a strong and homogenous emission of Cherenkov light in the water fronting the concave surface of the plaque. Despite a slightly lower light intensity at the periphery of the plaque, corresponding with the annular inactive rim, the light spread somewhat beyond the perimeter of the plaque (Fig. 2B). Second, a CCB plaque with an activity of 17.2 MBq was placed horizontally on the bottom of the acrylic box to enable side view imaging (Fig. 2C). In this setup, the Cherenkov light was clearly visible as a dome-shaped, luminous area reaching just beyond the rim and about 8 to 10 mm above the edge of the plaque (Fig. 2D). When visualized as a false-color coded image (Fig. 2E), the light intensity gradually decreased with increasing distance from the plaque, similar to the pattern seen in a two-dimensional dose distribution diagram of a CCB plaque with identical activity and radiation time (Fig. 2F).

### Clinical CLI of Uveal Melanoma Patients

Ten patients (4 men and 6 women) were included in the study. Nine patients were Caucasians and one patient was of East Asian ethnicity. The right eye was involved in six patients and the left eye in four patients. The mean age at the time of examination was 64 years (range, 39–80 years). All tumors were dome-shaped and moderately elevated, with a mean largest basal diameter of 9.7 mm (range, 5.8–13.0 mm) and a mean height of 3.1 mm (range, 2.0–4.6 mm). Two tumors were weakly, four were moderately, and two were markedly pigmented; two tumors were partially pigmented. The mean plaque activity was 0.056 MBq/mm^2^ (range, 0.035–0.089 MBq/mm^2^) at the time of examination, which took place at a mean of 2 days after plaque implantation (range, 1–4 days). The case characteristics are presented in the Table.

**Table.** Case Characteristics, Brachytherapy Parameters, and Results of CLI

				Tumor Dimensions, mm		Distance, mm				Plaque Activity		CLI				
Case No.	Age, years	Sex	Eye	Diameter	Height	Fovea	Optic Disc	Pigmentation	Ru-106 Plaque Type	MBq	MBq/mm^2^	Days after Surgery	Camera (Andor iXon)	600 nm Short-Pass Filter	Image Quality	Plaque–Tumor Relation
1	60	F	OD	8.5	2.8	0	1.7	Partial	CCB	10.4	0.035	3	DV887	No	Medium	Displaced inf
2	66	M	OD	8.2	3.5	3.8	2.0	Marked	CCB	19.1	0.064	2	DV887	No	Medium	Tilted inf
3	74	M	OS	10.2	2.0	0	3.5	Marked	CCB	17.7	0.060	1	DV887	No	High	Tilted sup
4	71	M	OS	9.5	2.0	1.7	2.2	Partial	CCB	17.5	0.059	1	DV887	No	High	Displaced temp
5	80	F	OD	5.8	2.5	0	3.0	Moderate	CCB	14.6	0.050	2	DV887	No	Low	Nongradable
6	60	M	OS	7.8	2.3	6.9	1.7	Moderate	CCA	7.0	0.048	1	DV887	No	Low	Nongradable
7	39	F	OD	13.0	4.0	2.5	1.7	Weak	CCB	11.4	0.038	4	Ultra 897	Yes	Medium	Centered
8	66	F	OD	10.0	2.9	2.7	5.4	Moderate	CCA	12.8	0.089	1	Ultra 897	Yes	Medium	Centered
9	53	F	OD	12.9	4.6	5.0	8.5	Moderate	CCB	21.8	0.073	2	Ultra 897	Yes	High	Displaced inf
10	69	F	OS	11.0	3.9	1.7	5.2	Weak	CCB	14.0	0.047	3	Ultra 897	Yes	High	Centered

F, female; inf, inferiorly; M, male; MBq, megabecquerel; OD, right eye; OS, left eye; Ru-106, ruthenium-106; sup, superiorly; temp, temporally.

Along with increasing experience, the methods for CLI were slightly modified during the study period. The red fixation LED combined with the integrated short-pass filter was used in the latter part of the study to improve the image quality. Further, the exposure time had to be individually adjusted according to both the plaque activity and the degree of tumor pigmentation. Weakly pigmented tumors required shorter exposure times to delineate the tumor area and, in some cases, a reference fundus image was needed to assess the plaque position in relation to the tumor. The level of impulse noise, leading to so-called salt-and-pepper pixels in the images, varied and was generally higher in images of patients treated with low activity plaques requiring long exposure times and a binning mode of 2 × 2.

In all patients, CLI revealed a circular area of light corresponding to the size and location of the ruthenium-106 plaque. Because the anterior tumor component was located outside the camera system's field of view, only the posterior part of the plaque and tumor were included in the images. In most cases, the tumor could be seen as a rounded, faintly darker area surrounded by a halo of Cherenkov light.

**Figure 3.**
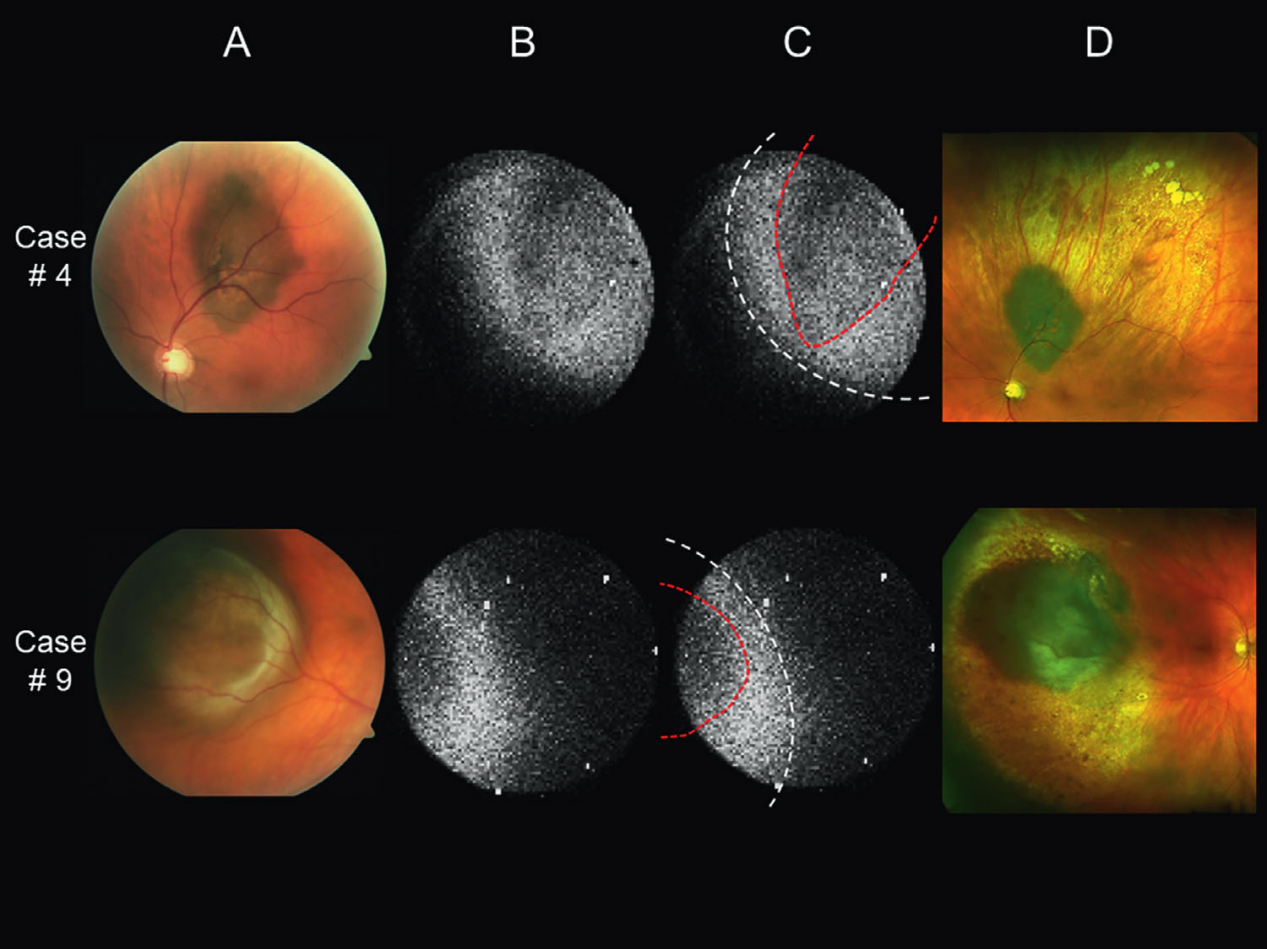
Fundus images of patients with posterior uveal melanoma. (A) Fundus photographs taken before ruthenium-106 plaque brachytherapy. (B) Cherenkov luminescence images taken after implantation of the ruthenium-106 plaque. (C) The same images as in (B) where the dashed white lines indicate the supposed plaque margin, and the dashed red lines delineate the area where the light is attenuated by the pigmented tumor. (D) Ultra-widefield fundus images taken during follow-up to assess the actual plaque position based on the radiation induced chorioretinal scar formation. The case numbers correspond to those listed in Table. In case 4, the plaque seems slightly decentered temporally relative to the tumor. In case 9, the plaque is located somewhat inferior to the tumor.

**Figure 4.**
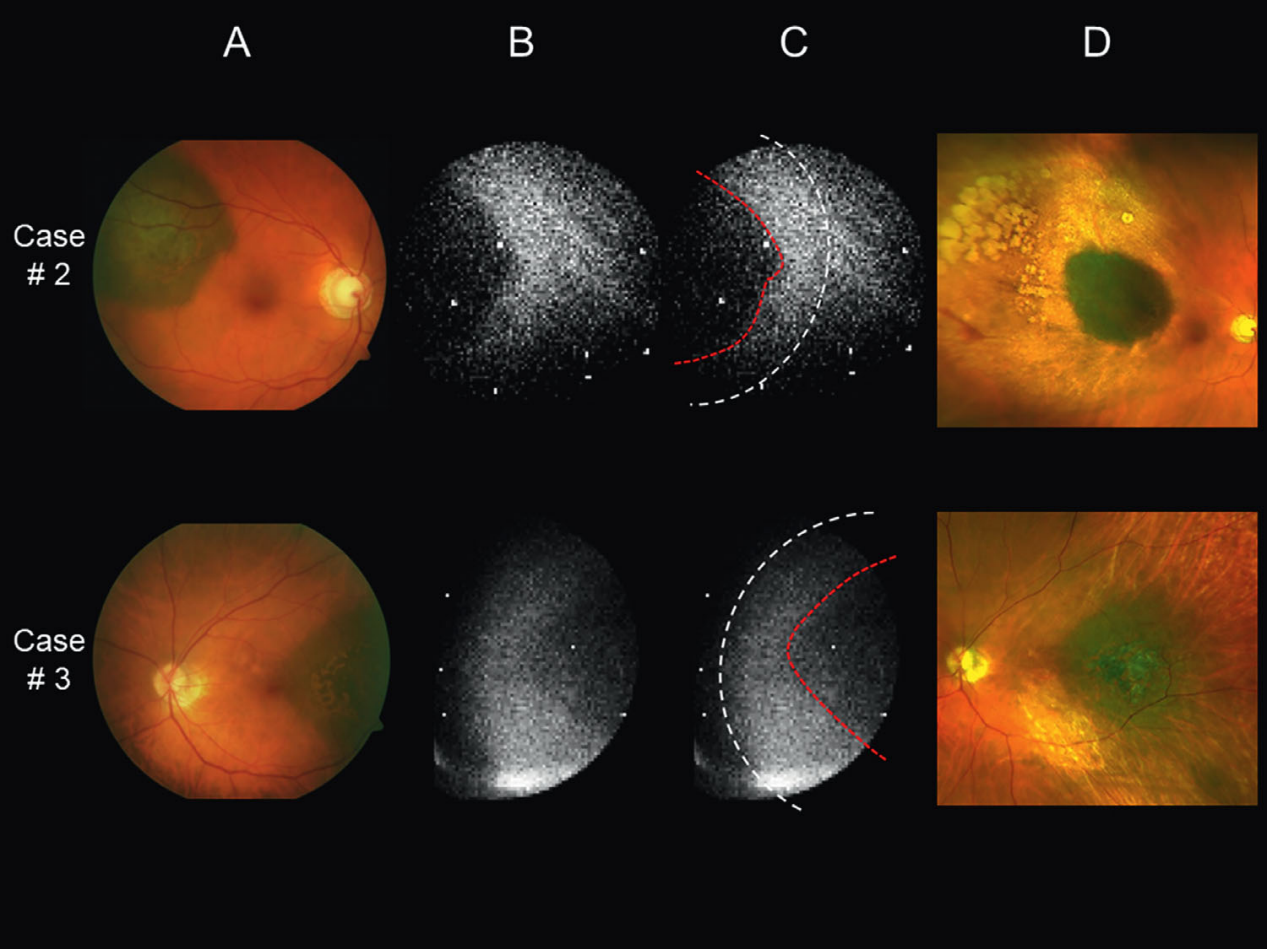
Fundus images of patients with posterior uveal melanoma. The image details (A−D) are as described in Fig. 3. The case numbers correspond to those listed in Table. In cases 2 and 3, the uneven intensity distribution of the Cherenkov light indicates a slight inferior and superior plaque tilt, respectively. Note the less prominent chorioretinal scarring inferior to the tumor in case 2 and superior to the tumor in case 3 (D).

In two patients (cases 5 and 6), only the ruthenium-106 plaque, and not the tumor itself, could be identified in the CLI images. In eight patients, the image quality was sufficient to assess the position of the plaque relative to the tumor (Table). In five of these patients, the plaque seemed to be somewhat displaced. In case 4, the plaque was slightly decentered temporally relative to the tumor, and in case 9, the plaque was located somewhat inferior to the tumor (Figs. 3A−C). An uneven intensity distribution of the Cherenkov light was present in case 2 and 3, indicating a mild inferior and superior plaque tilt, respectively (Figs. 4A−C). These assumptions were later confirmed by examining the extent and degree of the chorioretinal scarring seen in the follow-up ultra-widefield fundus photographs (Figs. 3D and 4D). Despite the slight displacement of the plaques shown during treatment, they adequately covered the tumor base. All tumors included in the study regressed as expected after ruthenium-106 brachytherapy, observed during a mean follow-up period of 13 months (median, 13.5 months; range, 1−20 months).

The imaging procedures were generally easy to carry out in clinical practice. Except for some tiredness during long-exposure imaging, none of the patients experienced discomfort or any negative side effects during or after the examination.

## Discussion

In this feasibility study, we have shown that CLI can be performed in vivo as a novel method to evaluate and document the position of ruthenium-106 plaques during brachytherapy of posterior uveal melanoma. By using an integrated EMCCD and fundus camera, we were able identify the plaque in the fundus of all the imaged patients. In most cases, it was also possible to assess the position of the plaque relative to the tumor, which appeared as a slightly darker area encircled by the Cherenkov light. By looking at the symmetry and intensity of the light distribution around the tumor, assumptions could be drawn about possible plaque dislocation, which was later confirmed by postoperative fundus photography.

To take advantage of the CLI technique, a number of difficulties had to be surmounted. In particular, the patients’ ability to fixate and keep their eyes still during long exposure imaging was crucial to achieve sufficient image quality. The use of a fixation light combined with an integrated short-pass filter to decrease the amount of ambient light proved to be successful in this regard. Although the diffuse plaque boundaries in the Cherenkov luminescence images may be due to unintentional eye movements, it is probably to a greater extent caused by the relatively strong light scattering within the eyewall, especially in the scleral tissue.^22^ Another significant limitation of our technique is that only a small part of the fundus is included in the Cherenkov luminescence images, making it impossible to determine the position of the entire plaque. However, this problem may be solved by using a fundus camera with a wider field of view combined with a larger sized EMCCD sensor.

Heterogeneous tumor pigmentation was also a challenge when analyzing the Cherenkov luminescence images. Because melanin absorbs light at a wide range of wavelengths,^23^ variations in melanin content may lead to an emission pattern that differs from the shape of the tumor. Likewise, possible differences in tumor vascularization and the amount hemoglobin will affect the light emission owing to high absorption in the blue–green wavelength range.^24^^,^^25^ The exposure time was therefore adjusted based on the degree of tumor pigmentation, because weakly pigmented tumors required shorter exposures for visualization. In cases where the tumor itself could not be identified, a comparable reference image taken with the same camera setup and the same field of view in low light conditions was useful to display the tumor and judge its location in relation to the plaque. Because there is a linear relationship between the level of radioactivity and the Cherenkov radiation,^20^ the necessary exposure time was also adjusted according to plaque activity at the time of imaging.

The experimental CLI of the ruthenium-106 plaques in water provided some interesting features of the Cherenkov radiation. The emission was homogeneously distributed over the entire concave plaque surface, and the light spread somewhat beyond the borders of the plaque both in the front and side view images. Most remarkable, when viewed from the side, the Cherenkov light appeared as a dome-shaped, luminous area above the plaque. Because the plaque was immersed in distilled water, which is transparent with low absorption and scattering coefficients,^26^^,^^27^ this phenomenon illustrates that the light is generated within the water itself and not projected from the plaque surface. The similarity between the false color-coded light intensity image and the regular dose distribution diagram raises the question of whether it is possible to determine the absorbed radiation dose from the Cherenkov luminescence. As demonstrated in previous studies, such an approach necessitates rigorous camera calibrations and dose distribution calculations.^28^^,^^29^

In the present study, CLI was performed to assess and document ruthenium-106 plaque placement during the postimplantation period. The main advantage of CLI over other imaging modalities such as B-scan ultrasound examination is that it provides an en face view of the tumor and the plaque, which makes it easier to determine the degree and direction of plaque displacement. CLI may in principle also be used intraoperatively to guide and monitor implantation of the plaque. However, this technique requires new technical solutions to integrate the EMCCD with the surgical microscope and to ensure absolute darkness around the operating field. In addition to ruthenium-106, other radioisotopes such as iodine-125 and palladium-103 are commonly used for episcleral brachytherapy. In tissues with a refractive index of 1.4, the electron energy threshold for Cherenkov radiation is 0.219 MeV.^30^ Ophthalmic plaques containing iodine-125 or palladium-103 will not induce Cherenkov light because these radioisotopes emit gamma photons with energies below the Cherenkov threshold in tissue.^29^^,^^31^

In conclusion, the feasibility of in vivo CLI imaging using an integrated EMCCD and fundus camera has been demonstrated in a small group of patients undergoing ruthenium-106 plaque brachytherapy for posteriorly located choroidal melanoma. The overall image quality differed markedly between the patients, and the best images were obtained when CLI was performed along with the use of a weak fixation light and the integrated 600 nm short-pass filter in patients with moderately to markedly pigmented tumors treated with high activity plaques. Further refinement of the CLI technique and the use of more sensitive cameras with a wider field of view are needed to improve its clinical usefulness, which will be explored in future studies.
